# The New Bharatiya Nyaya Sanhita Laws: Progress or Pitfall for Doctors?

**DOI:** 10.7759/cureus.69925

**Published:** 2024-09-22

**Authors:** Satvik N Pai, Madhan Jeyaraman, Naveen Jeyaraman, Sankalp Yadav

**Affiliations:** 1 Department of Orthopedics, People's Education Society (PES) University Institute of Medical Sciences and Research, Bengaluru, IND; 2 Department of Orthopedics, South Texas Orthopedic Research Institute (STORI), Laredo, USA; 3 Department of Clinical Research, Virginia Tech India, Dr. M.G.R. Educational and Research Institute, Chennai, IND; 4 Department of Orthopedics, ACS Medical College and Hospital, Dr. M.G.R. Educational and Research Institute, Chennai, IND; 5 Department of Medicine, Shri Madan Lal Khurana Chest Clinic, New Delhi, IND

**Keywords:** bharatiya nyaya sanhita, indian doctors, indian penal code, medical negligence, medical professionals

## Abstract

The Indian Penal Code (IPC), a relic of British colonial rule, was recently replaced by the Bharatiya Nyaya Sanhita (BNS) in July 2024. While the overhaul of the IPC was largely welcomed, it has sparked significant concern among the medical community, primarily due to Section 106 of the BNS. This section mandates imprisonment for doctors involved in deaths caused by rash or negligent acts during medical procedures, which many in the profession fear could lead to a climate of fear and hesitancy in performing critical medical interventions. This article delves into the contentious aspects of the BNS, comparing them to the old IPC provisions and examining the specific fears and protests of medical professionals. It also explores the legal nuances and potential impacts on medical practice, aiming to provide a balanced perspective and foster informed discussions within the medical community. Through this examination, the article seeks to dispel unfounded fears, highlight the protective measures within the BNS, and propose constructive approaches to ensure fair assessment in medicolegal cases, ultimately advocating for a legal environment that supports rather than hinders the medical profession.

## Editorial

The Indian Penal Code (IPC) was the official criminal code of the country until recently. It serves as a comprehensive legal framework for addressing criminal offenses in the country. This means it outlines the definitions of various crimes and prescribes the appropriate punishments. The IPC was inherited from British India and adopted by the Indian Republic after independence. Many of the laws in the IPC were outdated and plagued with colonial undertones and principles. In December 2023, the parliament passed a bill to replace the IPC with the Bharatiya Nyaya Sanhita (BNS). The BNS came into effect on July 1, 2024. The move to replace the IPC with BNS was predominantly perceived as a welcome move. However, a specific segment of the society, namely doctors, expressed significant displeasure with BNS. Medical associations across the country have been protesting against certain provisions of the BNS [1]. Social media is filled with messages expressing disappointment, fear, and apprehension among the medical community. In this article, we review these contentious sections of the BNS about doctors, analyze the changes made by the IPC, and provide a comprehensive overview of what the situation means for doctors in India. The aim is to create awareness, dispel unfounded fears, and enable medical professionals to discuss the matter with the correct perspective and understanding of this new law.

The big debating point: section 106 of BNS

Let us dive straight into what all of the outcry has been about. The entire controversy is predominantly based on one section in the BNS, i.e., Section 106. This section deals with the causing of death of an individual due to a rash or negligent act. The act states that the punishment for such an offense will be imprisonment for up to five years and shall also be liable to a fine. It then goes on to also say that if such an act is done by a doctor while performing a medical procedure, the punishment will be imprisonment for up to two years and is also liable for a fine [2].

Why have doctors and medical associations raised concerns against it?

Many doctors have raised their concerns about this section. As it makes the crime punishable with compulsory imprisonment, they believe this would make doctors fearful of undertaking surgeries or treating sick patients. The Indian Medical Association (IMA), the largest represented organization of doctors in India, has expressed their feelings of betrayal at the BNS. During the discussion on the new bill in parliament, Home Minister Amit Shah expressed their intent to differentiate between such crimes and the deaths occurring during medical treatments. However, they believe that this section of BNS does the opposite of that and has increased the punishment for doctors compared to earlier. Many medical associations across the country have voiced their protest against this section in the media, stating that this feels like the targeting of the medical fraternity. Many object that no other specialty faces this specific clause and question why doctors have been singled out for such a harsh punishment. The Ministry of Health and Family Welfare has responded to these protests in a press conference where they have clarified that the IPC, too, had a similar punishment, and Section 106 of the BNS is only a minor modification of the already-existing Section 304(A) of the IPC [3].

What has changed?

To understand both sides of this debate, we first have to understand what change in the law triggered this controversy. Section 106 of the BNS is constituted from Section 304(A) of the IPC, both dealing with the punishment for causing death by a rash or negligent act not amounting to culpable homicide [4]. These are the changes made from the IPC to BNS for this clause (Table 1).

**Table 1:** Changes between IPC 304 (A) and BNS 106 IPC: Indian Penal Code; BNS: Bharatiya Nyaya Sanhita

Components	IPC section 304 (A)	BNS section 106
Punishment for the offence	Imprisonment or fine	Imprisonment with or without a fine
Imprisonment duration	Two years	Up to five years
For doctors	The same as everyone (two years)	Less for doctors: up to two years; others: up to five years

The punishment in the IPC was the same for all persons, including doctors. The punishment was imprisonment for up to two years or a fine. In the BNS, the punishment has been made more severe. Imprisonment is now mandatory, and the duration of imprisonment has increased from up to two years earlier to up to five years now. The punishments have thus clearly been made more severe. However, they have now differentiated between the offense done by others and when such an incident happens by a doctor while performing a medical procedure. The punishment for doctors is less severe than for other persons, with imprisonment being for a maximum duration of two years, compared to five years for everyone else.

What are we talking about exactly here?

First, let us clarify what the offense in question is. This section deals with the causing of death by a rash or negligent act. Therefore, we are only discussing cases where a person has lost their life. So, there is no question of this section being applicable in cases of medical negligence where there is damage caused, but the death of the patient has not occurred. The next thing to remember is that every death occurring during a medical procedure will not lead to this section being applicable. The fact that a rash or negligent act is what caused the death has to be proven beyond doubt in a court of law. The word negligent, used in this act, does not have the same standards as medical negligence. For a case involving medical negligence to be considered in this section, not only would the requirements of medical negligence need to be satisfied, but it would also need to be gross medical negligence to warrant criminal charges. Proving this lies with the prosecution, i.e., the patient's attendants filing the case. The standards for a doctor to be charged under this section have not changed from the previous IPC. Rarely, even in cases of established medical malpractice, doctors have seldom been charged with criminal negligence under this particular section.

Are there other laws relevant for doctors in BNS?

Several other sections in the BNS are beneficial in defending doctors in courts of law. Section 18 states that nothing is an offense if it is done accidentally, without criminal intention, and with proper care and caution. Section 19 states that nothing is an offense, even with the knowledge that it is likely to cause harm if done without criminal intention and in good faith to prevent or avoid other harm to the person. Section 26 stipulates that an act is not considered an offense if it is not intended to cause death, is done in good faith, and is carried out with the consent of the individual. According to Section 30, an action is not considered an offense if it is not intended to cause death, is done in good faith, and is carried out without the consent of the individual, provided that the person is incapable of giving consent. The illustrations mentioned in Sections 26 and 30 are specific examples of surgeons operating on patients. All of these sections can be used to defend medical professionals when facing lawsuits.

So, are the doctors being protected or targeted?

We think the new law attempts to protect doctors rather than target them. Doctors have always been liable to be punished for such an offense under IPC. While the overall punishment for the offense has been increased from the IPC to the BNS, the punishment for doctors has been kept to a lesser degree than for non-doctors. The Health Ministry has reiterated the same. To the individuals within the medical fraternity questioning why other professions are not facing such a punishment, we would like to point out that such an offense performed by any other professional is also within the ambit of the act and would be facing the higher punishment of imprisonment of up to five years. The fact that most other professions do not deal with life-and-death situations has to be accepted by medical professionals as a risk we take in our profession. The real question, we believe, is not if we are being safeguarded but rather if we are being safeguarded enough.

What is the IMA saying?

We could not find any official statement released by the IMA on its website. However, the President of IMA, Dr. R. V. Asokan, has given several interviews and statements to the media. He has been local about the IMA's disappointment with the new law. As per media reports, the IMA has demanded that the professional service of medical professionals be exempted from criminal prosecution [3]. It has been pointed out that Section 26 of the BNS should apply in all cases of alleged medical negligence, as the act would be done in good faith with the individual's consent. Dr. Asokan has repeatedly pointed out that doctors performing a medical procedure never intend to cause harm and, hence, must not face criminal prosecution [5].

The IMA is the largest organization of doctors in India; any statements or directives expressed by that body have profound significance to the medical community in this country. It is not yet clear whether the statements provided to the media are the official statements of the IMA or the personal opinions of its President. While the IMA and Dr. Asokan should be commended for always championing the cause of the medical fraternity and campaigning for our causes tirelessly, in this instance, we think some of the comments made to the media might not have been legally sound. As per the law, the absence of intent to cause harm does not dissolve oneself in all the consequences of their actions. The mere absence of intent does not mean one cannot be held guilty of a criminal offense. For example, if a person rashly driving a car runs over a pedestrian who loses his life in the accident, the driver of the car will be held criminally liable for his action despite having no intent to cause the death of the pedestrian. We are also skeptical about the likelihood of the IMA's demand for complete exemption of doctors from criminal prosecution being accepted by the legislature. This demand has been voiced repeatedly by the IMA over the last couple of years, and lobbying of members of parliament to accept that demand has seen several doctors and associations put in considerable effort. While it would be wonderful for doctors if accepted, we do not believe that the demand for complete immunity from criminal prosecution would ever translate into law. Providing doctors with complete immunity from criminal prosecution would, in effect, make us above the law to some extent. It would also open up a lieu of incidents where those with ulterior motives would misuse such a law. A simplification of the events of the last four years, since the BNS was begun to be drafted, has been that the medical fraternity asked for complete immunity from criminal prosecution; the legislature has instead provided for marginal leniency toward doctors in the BNS, and now the medical associations are again demanding complete immunity.

What do we think the medical fraternity must do?

Medical associations across the country are currently in a state of confusion as to what the next move must be. The IMA itself had initially planned a campaign against the new law, but there have been no further updates regarding it. We recommend that medical associations do nothing about the new laws in terms of protests. They may not be ideal, but they are a little lenient toward us compared to others. This by no means is enough to safeguard medical professionals, and there are many problems with the way the law deals with medical cases. However, the punishment for this offense may not be one of the primary things we need to be putting all our might behind. We think that it would be more prudent to rather focus our energies on trying to bring in a system for a more fair assessment of medical negligence cases. Considering the highly specialized nature of medicolegal cases and the intricacies and ambiguities involved, we recommend that there should be a legislative recommendation or law making it mandatory that the expert opinion of at least two medical professionals specialized in the field related to the case be obtained and taken into consideration during the legal proceedings of a medicolegal case. It is also imperative that not only should the opinion of these experts be sought by the court in writing but also that the defense be allowed to put forward the medical facts of the case to these experts and have them give an opinion of the represented facts of the case by the defense and submit their opinions on the same to the court of law. We believe it to be an injustice for medicolegal cases to be determined without this opportunity and for the verdict of a medical matter to be given by nonmedical professionals who would not wholly understand the medical facts of the case without entertaining a medical discussion on the facts of the case between medical professionals.
